# Predictive Factors of One-Year Mortality in a Cohort of Patients Undergoing Urgent-Start Hemodialysis

**DOI:** 10.1371/journal.pone.0167895

**Published:** 2017-01-03

**Authors:** Luciene P. Magalhães, Luciene M. dos Reis, Fabiana G. Graciolli, Benedito J. Pereira, Rodrigo B. de Oliveira, Altay A. L. de Souza, Rosa M. Moyses, Rosilene M. Elias, Vanda Jorgetti

**Affiliations:** 1 Nephrology Division, Medical School, University of São Paulo, São Paulo, Brazil; 2 Medicine Master Degree Program, Universidade Nove de Julho (UNINOVE), São Paulo, Brazil; 3 Nephrology Division, School of Medical Sciences, University of Campinas (UNICAMP), Campinas, Brazil; 4 Department of Psychobiology, Federal University of São Paulo (UNIFESP), São Paulo, Brazil; Istituto Di Ricerche Farmacologiche Mario Negri, ITALY

## Abstract

**Background:**

Chronic kidney disease (CKD) affects 10–15% of adult population worldwide. Incident patients on hemodialysis, mainly those on urgent-start dialysis at the emergency room, have a high mortality risk, which may reflect the absence of nephrology care. A lack of data exists regarding the influence of baseline factors on the mortality of these patients. The aim of this study was to evaluate the clinical and laboratory characteristics of this population and identify risk factors that contribute to their mortality.

**Patients and methods:**

We studied 424 patients who were admitted to our service between 01/2006 and 12/2012 and were followed for 1 year. We analyzed vascular access, risk factors linked to cardiovascular disease (CVD) and mineral and bone disease associated with CKD (CKD-MBD), and clinical events that occurred during the follow-up period. Factors that influenced patient survival were evaluated by Cox regression analysis.

**Results:**

The patient mean age was 50 ± 18 years, and 58.7% of them were male. Hypertension was the main cause of primary CKD (31.8%). Major risk factors were smoking (19.6%), dyslipidemia (48.8%), and CVD (41%). Upon admission, most patients had no vascular access for hemodialysis (89.4%). Biochemical results showed that most patients were anemic with high C-reactive protein levels, hypocalcemia, hyperphosphatemia, elevated parathyroid hormone and decreased 25-hydroxy vitamin D. At the end of one year, 60 patients died (14.1%). These patients were significantly older, had a lower percentage of arteriovenous fistula in one year, and low levels of 25-hydroxy vitamin D.

**Conclusions:**

The combined evaluation of clinical and biochemical parameters and risk factors revealed that the mortality in urgent-start dialysis is associated with older age and low levels of vitamin D deficiency. A lack of a permanent hemodialysis access after one year was also a risk factor for mortality in this population.

## Introduction

In recent decades, chronic kidney disease (CKD) has evolved as a global public health problem that affects 10–15% of the adult population [1]. Several factors have contributed to this scenario, including the high prevalence of obesity, systemic arterial hypertension (SAH), diabetes mellitus (DM) and the increased longevity of the population. In 2010, approximately 1.9 million patients worldwide were on dialysis [2]. Early diagnosis, better quality of dialysis treatment and the increased number of transplants have decreased the mortality of CKD patients. The United States Renal Data System (USRDS) showed that the mortality rate associated with hemodialysis (HD), peritoneal dialysis and transplant patients decreased by 28, 47 and 51%, respectively, over the past twenty years [2]. However, compared to the general population, mortality for all cases is 6.1 to 7.8 times greater in dialysis patients, especially during the first year of therapy, when approximately 22% of patients die [2,3]. Therefore, it is important to identify risk factors that are associated with CKD and contribute to this high mortality.

Cardiovascular disease (CVD) is one of these factors and is the leading cause of death in both the general population and in patients with CKD [4]. Declining renal function increases the prevalence of CVD. Traditional risk factors that contribute to the development of CVD, such as age, SAH, DM, obesity and dyslipidemia, are more prevalent in patients with CKD than in subjects with normal renal function [5,6]. In addition, CKD itself is associated with worse cardiovascular outcomes, such as ventricular hypertrophy [5]. Systemic inflammation and malnutrition contribute directly to the increased mortality and hospitalizations in patients with CKD and are mutually dependent [7]. Therefore, markers of nutritional status, such as serum creatinine, albumin, and cholesterol, are associated with mortality, as are inflammatory markers such as C-reactive protein (CRP) and interleukin-6 [8]. Disturbances of mineral metabolism (CKD-MBD) have also been described as risk factors contributing to mortality in patients with CKD [9] and include disorders in serum calcium (Ca), phosphorus (P), parathyroid hormone (PTH), 25-hydroxy vitamin D and fibroblast growth factor 23 (FGF-23) [10].

Finally, an important risk factor for mortality is late referral to a nephrologist [11]. Although current guidelines recommend early referral of patients with CKD to a specialist, patients are not evaluated by a nephrologist prior to starting dialysis in an emergency service [12]. Although a higher mortality rate has already been identified in this scenario, it is unclear whether the above-mentioned factors influence survival when patients begin dialysis under emergency conditions.

Thus, the aim of the present study was to evaluate the risk factors associated with mortality in a cohort of incident and urgent-start HD patients. We hypothesized that, besides all factors associated with high mortality in patients starting dialysis, among those on urgent-start, late referral to a nephrologist and the absence a permanent vascular access during the first year will configure as important risk factors for mortality.

## Patients and Methods

### Patients

We included all adult patients who were admitted to the emergency room with clinical signs and symptoms of uremia, biochemical changes and an immediate need for dialysis. These patients are referring to those with refractory acidosis, hyperkalemia, hypervolemia, and have started dialysis within 24 hours after admission.

We excluded renal transplant recipients, patients taking vitamin D, those already in a dialysis program, and patients who recovered renal function. Data were prospectively collected, and patients were asked to participate at the presentation. There was no patient who declined to participate. Between January 2006 and December 2012, 444 patients with renal failure and an indication for immediate dialysis were seen at the Emergency Service of the Hospital das Clinicas da Faculdade de Medicina da Universidade de São Paulo. Twenty patients were excluded because three were under 18 years of age, four regularly used vitamin D, four were kidney transplant recipients, three were already on dialysis and six had recovered renal function. The remaining 424 patients who met the inclusion criteria were included in this study and were followed for one year.

Clinical parameters such as age, gender, race, etiology of kidney disease, presence and type of vascular access for dialysis, major comorbidities and regularly used medications were obtained from the clinical records of the Emergency Services. Events that occurred during the first year of dialysis were documented, respecting the confidentiality of information. We considered smokers those patients with a current smoking habitus. Arterial hypertension was considered the etiology of renal disease based on information gathered from the chart and the use of antihypertensive medication. On admission, SAH was defined as long-term use of anti-hypertensive medication or a systolic blood pressure greater than or equal to 140 mmHg and a diastolic pressure greater than or equal to 90 mmHg, obtained from annotations during the Hospital admission, including the measurements before hemodialysis sessions.

For the diagnosis of DM, we used information from the medical record, laboratory test results and the use of medications such as oral hypoglycemic agents and/or insulin.

Patients with total serum cholesterol levels greater than or equal to 200 mg/dL or LDL cholesterol above 100 mg/dL and triglycerides greater than 150 mg/dL or patients who were taking lipid-lowering drugs were considered dyslipidemic [13,14]. Medical history of dyslipidemia was also considered for the diagnosis.

Patients with a history of angina, acute myocardial infarction, angioplasty or myocardial revascularization were considered to have coronary ischemia. Those patients with a history of stroke or transient ischemic attack or those who had undergone carotid endarterectomy were classified as having cerebrovascular disease.

When the medical records documented dyspnea, peripheral edema, jugular stasis, hepatomegaly, lung congestion detected on physical examination or chest X-ray, limitations regarding engagement in physical activities or the use of specific medications, patients were diagnosed with congestive heart failure (CHF). The diagnosis was not based on one isolate symptom or sign. Medication recorded in association with CHF included carvedilol and nitrate plus hydralazine.

Those patients taking antiarrhythmic medications, with electrocardiogram changes of rhythm and rate, were classified as having cardiac arrhythmia. We considered arrhythmia any frequent rhythm but bradichardia, tachycardia and isolated extra beats. By antiarrhythmic, we mean any drug used exclusively to treat rhythm disorder, particularly amiodarone.

Patients with a history of claudication, ulceration or amputation due to ischemia of the limbs or those who underwent peripheral revascularization were diagnosed with peripheral arterial disease (PAD).

Clinical classification of comorbidities was done at the study entry and also confirmed continuously during the Hospital admission. For the definition of exposures and confounders in this study, the patients were categorized based on a clinical assessment made by the nephrology team, recruiting all the variables mentioned in the previous section as the most important for the clinical evaluation.

### Biochemical determinations

Blood samples were collected before the first HD session. Serum levels of hemoglobin, hematocrit, creatinine, albumin, ionized Ca, P, total cholesterol, LDL cholesterol, HDL cholesterol, triglycerides, glucose, iron, ferritin, alkaline phosphatase, and CRP (immunoturbidimetric method) were determined using routine laboratory techniques. On the same day, serum/plasma was stored for further analysis of serum intact parathormone (chemiluminescence immunoassay, Siemens (DPC), Germany), serum 25-hydroxy vitamin D (radioimmunoassay, DiaSorin, USA), serum intact FGF-23 (ELISA, Kainos, Japan), and plasma N-terminal-pro-brain natriuretic peptide (NT-pro-BNP) (chemiluminescence immunoassay, Roche, Switzerland).

### Follow-up

Causes of death were obtained from the patients' death certificates. Patients submitted to kidney transplant were censored at the date of transplantation for survival analysis.

### Ethics statement

The study and its consent procedure were approved by our local ethics committee (Comissão de Ética para Análise de Projetos de Pesquisa, CAPPesq, HCFMUSP, approval number CAAE: 45163715.4.0000.0068) and all subjects provided written informed consent before participation.

### Statistical analysis

**Fig 1:**
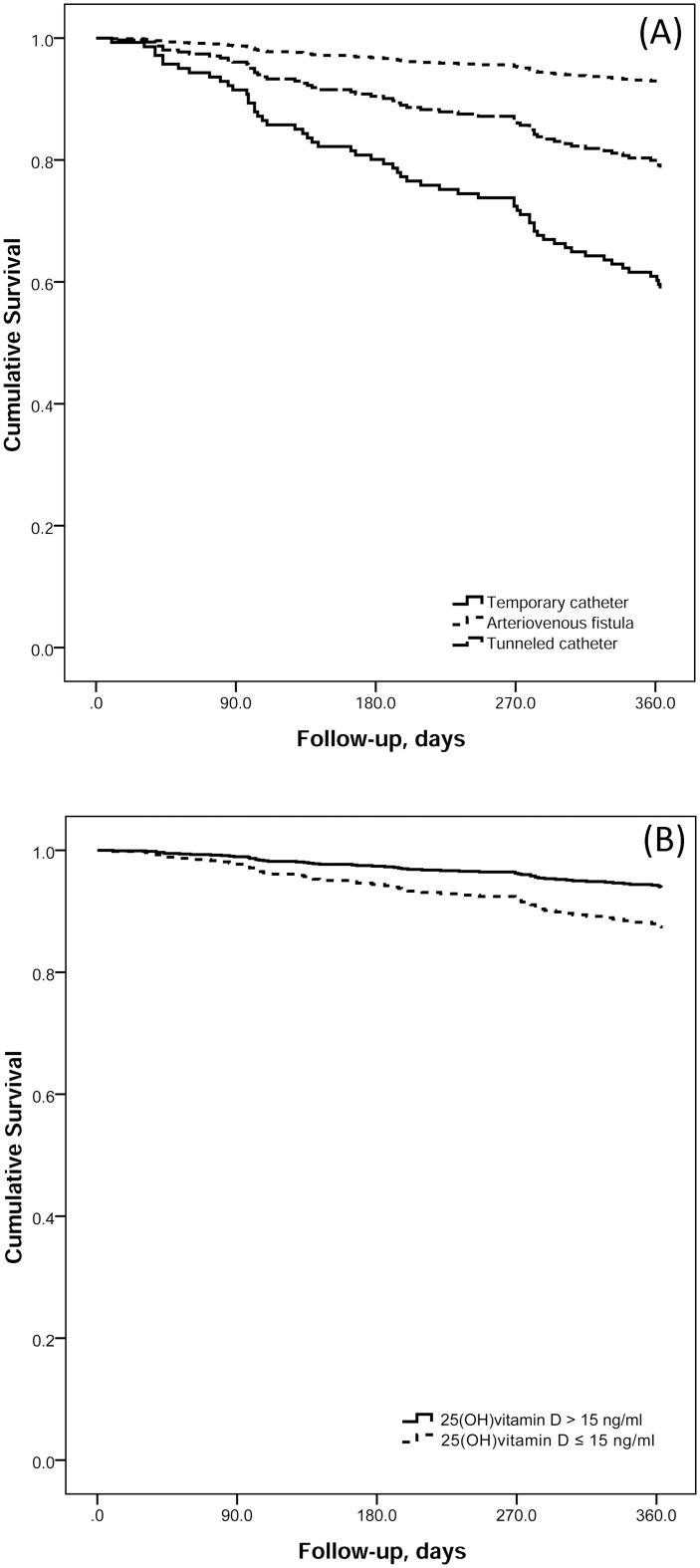
Cox regression-derived adjusted survival curves according to the type of vascular access (A), and levels of serum 25-hydroxy vitamin D (B). Model was the same stated in Table 3. (A). The continuous line represents patients with a temporary catheter, the upper dashed line represents patients with an arteriovenous fistula, and the middle dashed line represents patients with a tunneled catheter. (B). The continuous line represents patients with 25 hydroxy vitamin D levels > 15 ng/ml, and the dashed line represents patients with 25 hydroxy vitamin D ≤ 15 ng/ml.

Data was presented as the means ± standard deviations or medians (25th and 75th percentiles) depending on the skewness and adherence to a normal distribution of the data, as assessed by Shapiro-Wilk tests. To assess differences between survivors and non-survivors as a function of continuous dependent variables, independent t-tests or Mann-Whitney U tests were used. Chi-square contingency tests were performed to verify associations among surviving groups and outcome category variables analyzed in the study. Confidence intervals for the proportions using the normal approximation for the Binomial distribution were calculated. Based on the dependent variables that were significant in previous univariates tests, adjusted Cox regression of all-cause mortality with the candidate variables (age, sex, DM, DLP, dialysis vascular access, CHF, vitamin D) was performed and Confidence Intervals for the estimates of effect sizes (Hazard Ratio) were calculated using Breslow hazard function [15]. Survival plots for the significant outcomes related to mortality were also constructed and presented in Fig 1. For all analysis, a significance level of 5% (p < 0.05) was adopted. Analyses were performed with SPSS 20.0.1 software (SPSS Inc., USA)

## Results

### Baseline

Demographic and clinical characteristics of the study population categorized as survivors and non-survivors are shown in Table 1. Patients were relatively young, and most of them were men and Caucasian. SAH and DM accounted for more than 60% of the underlying cause of CKD. The main cardiovascular risk factors were smoking (19.6%); dyslipidemia (48.8%); CHF (19.8%); coronary ischemia (13.4%) and PAD (7.8%).

**Table 1:** Comparison between survivors and non-survivors.

	Survivors N = 364	Non-survivors N = 60	Total N = 424	p
Age (years)	48 ± 17	59 ± 18	50 ± 18	**< 0.0001**
Race (Caucasian)	251 (69)	42 (70)	293 (69.1)	0.871
Gender (male)	218 (60)	31 (52)	249 (58.7)	0.231
**CKD Etiology**				
Chronic hypertensive nephropathy	117 (32)	18 (30)	135 (31.8)	0.171
Diabetic nephropathy	104 (29)	21 (35)	125 (29.5)	
Glomerulonephritis	49 (13)	4 (7)	53 (12.5)	
Autosomal dominant polycystic kidney disease	11 (3)	1 (2)	12 (2.8)	
Other	83 (23)	16 (27)	99 (23.3)	
**Risk Factors**				
Smoking	69 (19)	14 (23)	83 (19.6)	0.428
Dyslipidemia	181 (50)	26 (43)	207 (48.8)	0.359
Congestive Heart Failure	66 (18)	18 (30)	84 (19.8)	**0.033**
Coronary ischemia	50 (14)	7 (12)	57 (13.4)	0.663
Peripheral artery disease	28 (8)	5 (8)	33 (7.8)	0.864
Arterial hypertension	349 (96)	57 (95)	406 (96)	0.754
Diabetes mellitus	126 (35)	24 (40)	150 (35)	0.419
**First vascular access**				
Temporary catheter	322 (88)	57 (95)	379 (89)	0.313
AVF	15 (4)	1 (2)	16 (4)	
Tunneled catheter	27 (7)	2 (3)	29 (7)	
**Final vascular access**				
Temporary catheter	8 (2)	10 (17)	18 (4)	**< 0.0001**
AVF	289 (79)	29 (48)	318 (75)	
Tunneled catheter	65 (18)	20 (33)	85 (20)	
PTFE	2 (1)	1 (2)	3 (1)	
**Medications at baseline**				
ACE/ARB	79 (22)	21 (35)	100 (23)	**0.025**
Beta-blocker	178 (49)	26 (43)	203 (48)	**0.424**
Calcium antagonist	247 (68)	34 (57)	274 (66)	**0.089**
Sevelamer	36 (10)	3 (5)	39 (9)	**0.225**
**Medications at the end of follow-up**				
ACE/ARB	84 (23)	21 (35)	105 (25)	**0.047**
Beta-blocker	177 (49)	26 (43)	203 (48)	**0.447**
Calcium antagonist	241 (66)	33 (55)	274 (65)	**0.092**
Sevelamer	247 (68)	31 (52)	278 (65)	**0.014**
Follow-up (days)	365 (76; 365)	188 (10; 363)	365 (10; 365)	**< 0.0001**

AVF: arteriovenous fistula; PTFE: polytetrafluoroethylene vascular access. ACE/ARB: angiotensin-converting enzyme inhibitors/angiotensin receptor blockers Data are expressed as the means ± SD, n (%) or medians (25, 75)

On physical examination upon admission, the mean systolic blood pressure was 152 ± 24 mmHg, and the mean diastolic blood pressure was 82 ± 15 mmHg. The majority of patients had hypertension (95.8%), and 86.8% were taking antihypertensive agents, most notably calcium channel blockers and beta-blockers. Of note, only 23% of patients were prescribed angiotensin-converting enzyme inhibitors/angiotensin receptor blockers (ACEs/ARBs). A considerable proportion of diabetics (32.8%) and individuals with dyslipidemia (48.7%) had not been treated with specific medications. Most patients had no vascular access on admission and required a temporary catheter (TC) implant (89.4%). Only 16 patients (3.8%) had an arteriovenous fistula (AVF), and were being followed by nephrologist.

Table 2 summarizes the laboratory data of survivors and non-survivors. In 83.7% of patients, serum hemoglobin was below 11 g/dL, ferritin was less than 100 mg/dL in 10.3%, and the transferrin saturation rate was less than 20% in 44.5% of patients, using the minimal cut-off preconized by guidelines. Levels below the reference range were found for albumin in 33.5%, total cholesterol in 70.9%, LDL fraction in 54.8%, and triglycerides in 51.8%. Only 24% of patients with total cholesterol levels higher than 200 mg/dL were receiving statins (p = 0.064). Most patients (79.9%) had elevated CRP levels (higher than 5.0 mg/L).

**Table 2:** Laboratory data at baseline comparing survivors and non-survivors.

Variable	Reference Range	Survivors n = 364	Non-Survivors n = 60	Total n = 424	p
**Creatinine**, mg/dL	**0.7–1.2**	**9.1 (6.6; 13.9)**	**7.8 (6.0; 10.5)**	8.8 (6.5; 13.3)	**0.014**
**Urea**, mg/dL	**10–50**	**215.5 (176; 275)**	**190.5 (158; 252)**	210 (173; 273)	**0.010**
**Hemoglobin**, g/dL	**12–17**	8.8 (7.7; 10.3)	8.4 (7.6; 9.6)	8.8 (7.7; 10.1)	0.220
**Hematocrit**, %	**37.5–49.5**	27.3 (23.6; 31.3)	26.3 (23.9; 29.8)	27.2 (23.8; 31.1)	0.459
**Ionized calcium**, mg/dL	**4.6–5.3**	**4.44 ± 0.62**	**4.68 ± 0.46**	4.60 (4.2; 4.9)	**0.005**
**Phosphorus**, mg/dL	**2.7–4.5**	**7.4 ± 3.5**	**6.2 ± 1.9**	6.7 (5.5; 8.4)	**0.011**
**Alkaline phosphatase**, U/L	**40–129**	**88 (70; 124)**	**100 (77; 134)**	90 (71; 128)	**0.029**
**25-OH vitamin D**, ng/mL	**30–100**	12.3 (8; 18)	12.4 (8.0; 15.0)	12.3 (8; 18)	0.346
**Parathyroid hormone**, pg/mL	**10–65**	171 (48; 392)	146 (46; 366)	165 (48; 386)	0.484
**FGF-23**, pg/mL	**< 53**	1,960 (672; 4,303)	1796 (77; 21093)	1,927 (614; 4,212)	0.380
**Triglycerides**, mg/dL	**< 150**	**151 (105; 212)**	**127 (403; 3821)**	147 (103; 207)	**0.031**
**Total cholesterol**, mg/dL	**< 200**	**177 (146; 205)**	**156 (129; 211)**	175 (145; 205)	**0.050**
**LDL cholesterol, mg/dL**	**< 130**	96 (74; 120)	93 (61; 125)	95.5 (73; 121)	0.296
**HDL cholesterol, mg/dL**	**> 60**	43 (35; 54)	42 (34; 57)	43 (35; 54)	0.965
**Albumin**, g/dL	**3.4–4.8**	3.26 ± 0.7	3.17 ± 0.6	3.3 (2.8; 3.7)	0.323
**C-reactive protein, mg/L**	**< 5.0**	**13.6 (5.7; 43.8)**	**32.2 (8.9; 107.0)**	14.9 (5.8; 47.5)	**0.012**
**Glucose, mg/dL**	**70–99**	89 (79; 135)	90 (79; 131)	89 (79; 133)	0.807
**Iron**, μg/dL	**59–158**	**53 (38; 77)**	**43 (23; 67)**	51 (36; 74)	**0.012**
**Ferritin**, ng/dL	**30–400**	346 (195; 628)	272 (154; 522)	336 (193; 616)	0.102
IBC, %	**20–50**	22.1 (14.9; 31.1)	19.0 (12.5; 30.1)	21.8 (14.7; 30.7)	0.178
NT-pro-BNP, pg/mL	**< 100**	4,793 (1,061; 14,691)	4,900 (1,194; 15,654)	4,839 (1,078; 14,813)	0.569

IBC: Iron-binding capacity; NT-pro-BNP: N-terminus of the prohormone brain natriuretic peptide. Data are expressed as the means ± standard deviations or medians (25, 75).

Hypocalcemia and hyperphosphatemia were found in 48.1 and 74.2%, respectively.

Vitamin D was below the reference range (25-hydroxy vitamin D levels < 30 ng/mL) in 95.1%, and secondary hyperparathyroidism (PTH greater than 65 pg/mL) was found in 69.1% of patients. FGF-23 levels were normal in 0.47% of patients, with a median of 1927 pg/mL (19.1 to 37,721 pg/mL).

NT-pro-BNP levels were measured in 399 patients, and only eight patients had serum levels within the reference range (up to 100 pg/mL). Forty-three percent of patients had levels above 6000 pg/mL, a cut-off value usually observed in CHF patients on dialysis [16]. Among these patients, CHF was more prevalent than among patients with levels below 6000 pg/mL (47.6% vs. 36.8%, respectively; p = 0.006).

### Follow-up

There was no loss to follow up. At the end of the first year, 60 patients had died (14.1%), and nine had received a kidney transplant (2.1%) (with a median follow-up time of 188 days). Twenty-seven patients (45%) died due to infection; twenty-seven patients (45%) died due to cardiovascular causes, including acute myocardial infarction, stroke and sudden cardiac death. Other causes such as pulmonary embolism, hypovolemic shock, and suicide accounted for the remaining causes of death. Patients who died were older, had CHF, and still remained without AVF. In addition, non-survivors had lower levels of urea, creatinine, total cholesterol, triglycerides and serum iron, while CRP levels were higher (Table 2). Regarding mineral metabolism markers, we observed that the levels of serum Ca and alkaline phosphatase were higher, while serum P was lower. In addition, non-survivors had a higher prevalence of serum levels of 25-OH vitamin D lower than 15 ng/mL (81.4% vs. 63.7%, respectively, p = 0.008). The mortality risk associated with these levels of vitamin D was 1.9 times higher in these patients.

Non-survivors were receiving more ACE/ARB at baseline (35 vs. 22%, p = 0.025) and also at the end of follow-up (35 vs. 23%, p = 0.047). Non-survivals were also receiving more anti-platelets drugs (45 vs. 33%, p = 0.029) at baseline, and less phosphate binder sevelamer at the end of follow-up (52 vs. 68%, p = 0.014) than survivors.

During the first year of HD, there were 112 hospitalizations, and the hospitalization rate was 264/1000 patients, which was higher among non-survivors (47 [95%CI 38.1–56.6] vs. 23% [95%CI 15.4–31.0] survivors, p = 0.0001). During the follow-up, these patients had a higher incidence of vascular access infection and lung infection (91.7% [95%CI 86.9–0.97] vs. 80.8% [95%CI 73.0–87.7], p = 0.04 and 80% [95%CI 72.0–86.9 vs. 64% [95%CI 55.4–73.2], p = 0.017, respectively). In addition, patients who died developed CHF (p = 0.033), arrhythmias (p = 0.008), coronary ischemia (p = 0.018) and PAD (p = 0.026).

To identify factors associated with mortality, we conducted a Cox regression analysis (Table 3). In the model, we included all significant variables defined as a p-value less than 0.05 from the univariate analysis in addition to variables that are consider as confounders for mortality, such as age, gender, presence of DM and CHF. Risk factors associated with mortality within one year included age, CHF, absence of arteriovenous fistula at follow-up, dyslipidemia and levels of 25-hydroxy vitamin D lower than 15 ng/ml. Fig 1 shows the adjusted mortality curves derived from the Cox analysis for Vascular access type (Fig 1A) and levels of 25-hydroxy vitamin D (Fig 1B).

**Table 3:** Cox regression analysis of factors associated with mortality.

Risk factors	β	SE	HR	95% CI		p
				Lower	Upper	
**Age**	**0.033**	**0.008**	1.034	1.017	1.051	**0.0001**
**Male gender**	0.154	0.267	1.167	0.691	1.969	0.564
**Congestive heart failure**	0.333	0.297	1.396	0.780	2.497	0.261
**Vascular access**						
**Temporary catheter**	Reference					**0.0001**
**AVF vs. Temporary catheter**	**-1.944**	**0.373**	0.143	0.069	0.298	**0.0001**
**Tunneled catheter vs. Temporary catheter**	**-0.852**	**0.401**	0.457	0.194	0.936	**0.034**
**Diabetes**	**0.160**	**0.272**	1.173	0.689	1.998	0.557
**Vitamin D < 15 ng/ml**	**0.759**	**0.339**	2.136	1.098	4.156	**0.025**

## Discussion

In this study, we prospectively followed 424 incident patients with CKD stage 5 who started HD in the emergency service and were followed for one year. The significant findings were as following: Except for 16 patients with an AVF (3.8%), the majority of patients were not being followed-up by a nephrologist; SAH and DM accounted for most cases of kidney disease. Absence of an AVF after one-year follow-up accounted for mortality as well as indirect evidence of malnutrition (low serum levels of P, cholesterol and 25-hydroxy vitamin D).

Incident HD patients have high mortality rates [17]. In the current study, the clinical conditions of the patients on admission were of concern because most were hypertensive; had no definitive vascular access; were anemic; had evidence of abnormal mineral metabolism; were not adequately treated for DM; and had hypertension, dyslipidemia, high serum CRP levels and indirect signs of malnutrition. Altogether, this clinical scenario reveals a failure in the primary health care system. Nonetheless, the mortality rate was still low compared with other studies [18]. The access to a major complexity hospital and the quality of HD offered during the follow-up, may explain, at least partially, the observed mortality rate.

Recently, a review by Cochrane Database of Systematic Reviews analyzed the results of 40 studies with 63,887 patients, of whom 43,209 were referred for early follow-up with nephrologists, and 20,678 were referred at a later stage [19]. The results showed reduced mortality and hospitalization rates and more patients with vascular access for HD in those with early referrals to a specialist. In our study, late or no referral to a nephrologist is a plausible explanation to the absence of HD vascular access, while international records suggest that at least 50% of patients starting HD should have an AVF. We have demonstrated that having an AVF during the first year of HD was a protective factor. However, we cannot guarantee that these patients had a TC placement, even though for a short period of time.

Malas et al. conducted a retrospective study using data from the American Registry Data of the USRDS in which they evaluated 510,000 patients [20]. Most patients (82.6%) started HD with a TC, and only 14% of the patients had an AVF. In agreement with our findings, AVF was associated with a lower mortality rate (23%). An AVF can reduce mortality as it is related to early referral to nephrologists and vascular surgeon and anatomical conditions for a fistula creation. On the other hand, our study can be biased due to higher probability of obtaining an AVF for persons who survive longer, and not necessarily means that having an AVF can improve survival.

At the end of the one-year follow up, 75% of our patients had an AVF, which has a positive impact on mortality, primarily compared with a TC. Interestingly, a tunneled catheter was associated with a survival curve that is not necessarily similar to an AVF. This finding may reflect either the clinical condition of the patients or the dialysis center to which they were referred.

Infections related to vascular access are the most common identifiable source of infection in patients on HD that contributes to increased mortality [21]. In addition to increased mortality, the direct and indirect costs of hospitalizations are high. In our country, this cost is unknown, but data from the literature support a range from 17,000 to 32,000 dollars per patient [22].

Most of our patients (80%) had hemoglobin levels lower than 11 g/dL. Although we did not detect an impact of anemia on mortality, this factor is considered a non-traditional risk factor for the development of CVD and contributes to myocardial hypertrophy and higher mortality in patients with CKD [23]. We did not detect an impact of lipid disorders either; these disorders are a risk factor that increases the mortality of both the general population and patients with CKD. The lipid profile of the patients suggests the presence of malnutrition as 50% had low levels of cholesterol those who did not survive had lower serum triglycerides, urea, creatinine, P and iron levels and high serum levels of CRP, all of which are indirect effects of malnutrition. Unfortunately, we have no data regarding body weight, height, or body mass index or information about recent weight loss, dietary recall, gastrointestinal symptoms and functional capacity to confirm the presence or the absence of malnutrition. However, protein-calorie malnutrition associated with inflammation has been extensively studied in recent years and is considered a risk factor that increases the mortality of patients with CKD [24].

Regarding the mineral metabolism disorders that were present in most patients, serum Ca levels and alkaline phosphatase levels were higher in patients who died, which might reflect increased bone turnover, whereas the low levels of P reflects malnutrition [25].

Levels of 25-hydroxy vitamin D lower than 15 ng/mL doubled the risk of death independently. Vitamin D deficiency is highly prevalent in patients with CKD, particularly in those on CKD stage 5, and is associated with various clinical outcomes, such as doubling serum creatinine levels in patients with kidney disease worsening of anemia, muscle weakness, vascular calcification, endothelial dysfunction and cardiovascular events [26]. Serum levels of 25-hydroxy vitamin D are influenced by various factors such as seasonality, physical activity, inflammation, DM and proteinuria, which could not be analyzed in this study. Recently, our group evaluated the levels of 25-hydroxy vitamin D in normal subjects during the winter and summer, and the values were 22 ng/mL and 34 ng/mL, respectively. These values were higher than that those observed in the patients in this study, which was 12.3 ng/mL. The definition of hypovitaminosis varies among researches and organizations, and the best cutoff to predict mortality among patients with CKD is still debatable. We have found that levels lower than 15 ng/ml could predict mortality, in agreement with other published studies, reinforcing the idea that vitamin D deficiency increases the risk of death [27]. However, whether vitamin D replacement improves survival in incident patients on HD requires further investigation.

In recent years, many publications have demonstrated that serum levels of FGF-23 are associated with increased mortality, renal disease progression and development of CVD. This hormone promotes cardiomyocyte hypertrophy [28]. We found no association between mortality and FGF-23. Serum levels of FGF-23 were very high in virtually all patients, making it difficult to detect any difference and decipher its role in factors related to survival.

Non-survivors commonly took the antihypertensive ACE-ARB and antiplatelet agents, which was probably due to their more severe clinical conditions.

The protective effects of the P binder sevelamer hydrochloride on survival of patients on dialysis have been previously described and were also observed in our study. In addition to reducing harmful P levels, sevelamer hydrochloride also lowers cholesterol levels, FGF-23, and advanced products of glycosylation (AGEs), and reduces Ca overload produced by Ca-based binders [29]. Sevelamer, however, may be associated with nutritional status or access to a better quality health care, instead of being a predictor of survival.

In this study, the life expectancy of patients with CHF was slightly lower than that of patients who did not have this complication. In the American population, CHF is the leading cause of hospitalization in patients over 65 years, with an annual incidence of approximately one million individuals and a mortality rate of 300,000 patients/year [30]. The association between CKD and CVD is well established. The interaction between the two systems triggers pathological processes and accelerates dysfunction in both disorders, which is referred to as cardiorenal syndrome [31]. As clinical diagnosis was made in retrospect based on patient files, it is plausible that some information may be lacking from source data available to the assessor, such as exercise intolerance and chronic obstructive pulmonary disease. Also, fluid retention may be due to renal failure rather than CHF.

Even without properly analyzing the nutritional status of patients, we can infer that malnutrition favors mortality, as evidenced by the protective effect of higher levels of urea and triglycerides. The lowest serum levels of urea and cholesterol were associated with higher mortality in patients with CHF.

In summary, patients who started dialysis at emergency room, malnourished and who have no AVF during the first year of dialysis are at higher risk of mortality. Efforts should be made to early referral to a nephrologist. Vitamin D levels lower than 15 ng/ml in these patients also predict 1-year mortality. However, whether supplementing such vitamin would decrease the mortality risk is still unknown.

## Supporting Information

S1 File Raw data of the study. (SAV)
